# Walkability and Fitness Center Prices, Opening Hours, and Extra Services: The Case of Madrid, Spain

**DOI:** 10.3390/ijerph17155622

**Published:** 2020-08-04

**Authors:** Jairo León-Quismondo, José Bonal, Pablo Burillo, Álvaro Fernández-Luna

**Affiliations:** Faculty of Sports Sciences, Universidad Europea de Madrid, Calle Tajo S/N, Villaviciosa de Odón, 28670 Madrid, Spain; jose.bonal@universidadeuropea.es (J.B.); pablo.burillo@universidadeuropea.es (P.B.); alvaro.fernandez2@universidadeuropea.es (Á.F.-L.)

**Keywords:** walkability, fitness centers, services, urban environment

## Abstract

Walkability has been associated with urban development and political plans, contributing to more connected cities with improvements in communication, shopping, and pedestrian base. Among these services, fitness centers are becoming important elements for communities due to their impact on the health and welfare of citizens. The present study aims to examine how an area’s Walk Score^®^ affects fitness center services, specifically membership costs, opening hours, and aquatic services. Data from 193 fitness centers were retrieved, representing all the areas of the municipality of Madrid, Spain, including fitness centers in the 21 city districts. A nonlinear relationship between an area’s Walk Score^®^ and fitness centers’ monthly fees is observed. Only in premium fitness centers, a weak curvilinear model is observed, following a quadratic equation, showing that fitness centers with higher prices are in less walkable areas. Additionally, the association between Walk Score^®^ and a fitness center’s opening hours reveals that fitness centers with wider hours of operation tend to be in moderately to highly walkable locations. Lastly, the existence of a swimming pool is related to a lower Walk Score^®^. Thus, fitness centers in less walkable areas try to offer additional services as differentiation from competitors, whereas centers in walkable locations use this advantage as a strength.

## 1. Introduction

The concept of walkability is defined as “the extent to which the built environment is friendly to people who walk to work, for leisure or recreation, to travel, for exercise, or to access services” [1]. Overall, it can be broadly understood as the extent to which an area, usually an urban area, is walking-friendly [2]. Proven benefits of living in walkable environments include a healthier lifestyle [3,4] and less polluted and congested streets [5,6] as well as economic benefits [7]. This evidence has led to an increase in research on the design of walkable cities [1]. Following previous work, there are some urban elements that are central for walkability. These are the connectivity of the path network, linkage with other modes of transport (bus, subway, or train), safety from both traffic and social crime, quality of path (width, paving, or signing) and path context (street design, visual interest, or landscape), and varied land use patterns (reaching most local services on foot within 10–20 min, including uses such as shops, cafes, banks, laundries, grocery stores, parks, or fitness centers) [5]. Thus, walkability is tightly associated with urban development and political plans, contributing to a more connected city with improvements in communication, services, shopping, and pedestrian base [8].

City services have been widely analyzed in recent years in relation to the concept of smart cities [9], user information and communication technologies [10], and the habits of individuals based on their relationship with the environment [11,12]. Among the different approaches, it is not easy to find a variable to measure the quality of life of citizens tightly related to the services that cities offer—with the exception of satisfaction surveys and consumption habits. Different perspectives on walkability also lead to diverse focuses [13]. Consequently, a wide range of quantitative and qualitative tools have been used to assess the walkability of urban environments. Some examples are the Pedestrian Environment Review System (PERS), Pedestrian Level of Service (POS), or Geographic Information Systems (GIS). In view of the range of studies, diverse issues in measuring walkability have also emerged [14,15], including trip purpose, temporal issues (different times of day), walking barriers, and the perceived quality of walkable spaces. These issues highlight the difficulties in assessing walkability. In this regard, following Vale et al. [15], there are four main methodological categories for assessing walking accessibility: distance-based, gravity-based, topological or infrastructure-based, and walkability or walk score-type. This research focuses on this last approach.

Since its creation in 2007, Walk Score^®^ has been one of the most widely used methods worldwide for walkability assessment. Walk Score^®^ is a United States-based company that provides walkability services and apartment search tools through a website and mobile applications. Walk Score^®^ uses data provided by the Google™ AJAX Search application program interface (API) through a geography-based algorithm [16]. The Walk Score^®^ algorithm calculates a score of walkability based on the distance to 13 categories of amenities (grocery stores, coffee shops, restaurants, bars, movie theaters, schools, parks, libraries, book stores, fitness centers, drug stores, hardware stores, clothing/music stores). Each category is weighted equally, and points are summed and normalized to yield a score of 0–100 [17].

The score has been validated by the scientific literature as a reliable way to measure access to walkable amenities mainly in the United States [18,19], and its use is being extended to other regions, including Europe [14,20,21] and Asia [22,23]. However, some researchers claim that Walk Score^®^ and other applications do not replace conventional street network measures but are complementary [24]. A recent systematic review indicates that the analysis of walkability using Walk Score^®^ is inconsistent due to research results tending to only partly support the validity of Walk Score^®^ [2]. Despite the criticism, the research community considers that there is no reason to believe that Walk Score^®^ is substantially different than in the United States-based studies [20,21], and it is valid in high population density urban areas [19].

In addition, Walk Score^®^ has been used in the scientific literature to evaluate physical activity, health status, and sedentary behavior [20,25,26], tourism accommodation and services [14,23], eating habits [27], and walking and gaming mobile apps use [28]. Nevertheless, there is no specific research about Walk Score^®^ and fitness centers, considering that gyms and sports centers are part of the Walk Score^®^ algorithm. This knowledge gap in the literature regarding fitness centers should be addressed, since these sports services are fundamental for active lifestyle promotion and have important impacts on health [29,30]. Furthermore, it is difficult to find previous research that links Walk Score^®^ with important service variables such as price and opening hours. As an example, other authors have found how Walk Score^®^ could affect pricing in other tourist services [23]. For that reason, this paper tries to answer the research question: “How does location affect fitness center services?”.

In this vein, several authors have raised the importance of the evaluation of fitness services, meaning a detailed analysis of the provided service that contributes to making managerial actions more precise. Additionally, authors have examined specific business models based on price [31] or the analysis of the importance and performance of different services and management decisions [32]. For this purpose, after the literature review, a possible relationship between the Walk Score^®^ and different variables such as prices, opening hours, and specific services at fitness centers is considered, depending on the city district and its walkability. Traditionally, price has been identified as one of the main reasons to enroll in a fitness center [32]. Moreover, opening hours are crucial, even more so in a big urban area such as a capital city. Additionally, extra services such as aquatic services contribute to attracting more people to the centers. Thus, this study aims to examine how the location, measured by Walk Score^®^, affects fitness center services through different variables such as membership costs, opening hours, and aquatic services in fitness centers in the municipality of Madrid.

## 2. Materials and Methods

**Figure 1:**
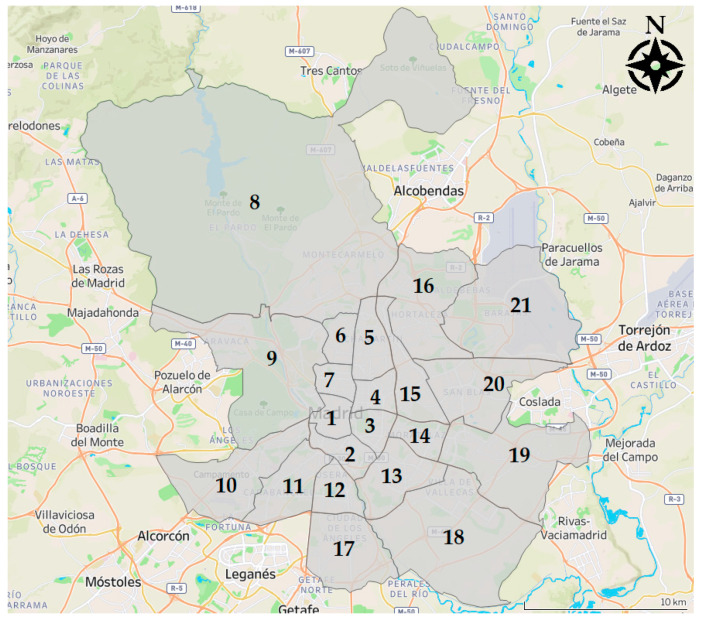
Districts in the city of Madrid: (1) Centro; (2) Arganzuela; (3) Retiro; (4) Salamanca; (5) Chamartín; (6) Tetuán; (7) Chamberí; (8) Fuencarral-El Pardo; (9) Moncloa-Aravaca; (10) Latina; (11) Carabanchel; (12) Usera; (13) Puente de Vallecas; (14) Moratalaz; (15) Ciudad Lineal; (16) Hortaleza; (17) Villaverde; (18) Villa de Vallecas; (19) Vicálvaro; (20) San Blas-Canillejas; (21) Barajas.

The sample is composed of 193 fitness centers (179 private, 14 public) located in the city of Madrid, Spain, covering the 21 districts of the municipality (Figure 1). Madrid, located in the center of the Iberian Peninsula, is the capital city of Spain and has a population of almost 3.3 million inhabitants. Madrid has a land area over 600 square kilometers.

The selected fitness centers were retrieved from Madrid Council’s Open Data Portal, which allows access to a complete database of the business census of the city. The June 2019 data package was used [33]. Several stages were addressed before reaching the final sample of 193 centers. Firstly, the complete business census was filtered by activity, obtaining the number of 730 sports centers, thus eliminating non-sports businesses. Secondly, those oriented to fitness activities were selected, as well as those currently inactive were deleted, obtaining 252 centers. Thirdly, for each of them, the monthly fee, business model (low-cost ≤ 30€; mid-market = 30€–60€; premium ≥ 60€), and the existence or not of a swimming pool were checked. Any center with a lack of any of the aforementioned information was deleted from the database, resulting in a final number of 193 fitness centers. Lastly, the Walk Score^®^ for each center location was manually derived from the website www.walkscore.com, using the exact address of the company [34].

For the analysis of the data, Walk Score^®^ was used as a categorical variable, instead of continuous, as recommended by previous work [23,35]. Therefore, four quartiles were established (≤90; 91–95; 96–98; ≥99). The data analysis was performed with IBM SPSS 23.0 Statistics software (IBM Inc., Chicago, IL, USA), including descriptive statistics, *t*-test, Mann–Whitney U test, and quadratic regression, as suggested in previous research [14,23]. The critical level of significance was set at *p* < 0.05.

## 3. Results

### 3.1. Descriptive Statistics

**Figure 2:**
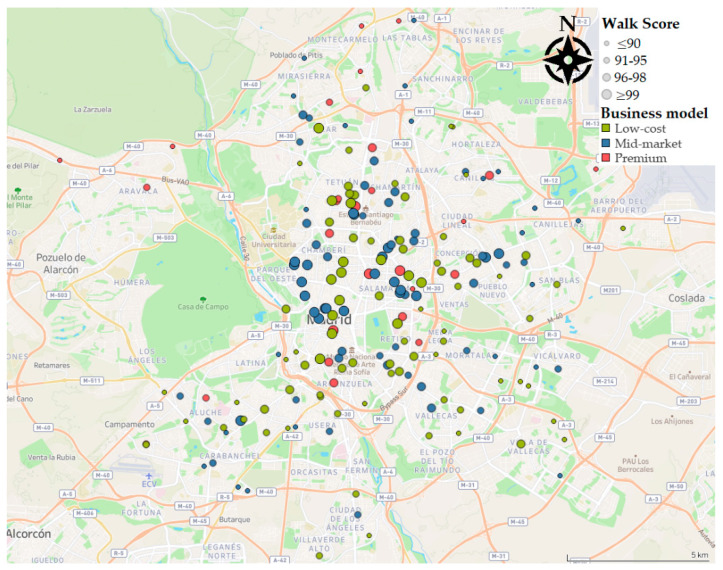
Distribution of the fitness centers in Madrid, sized by Walk Score^®^.

**Figure 3:**
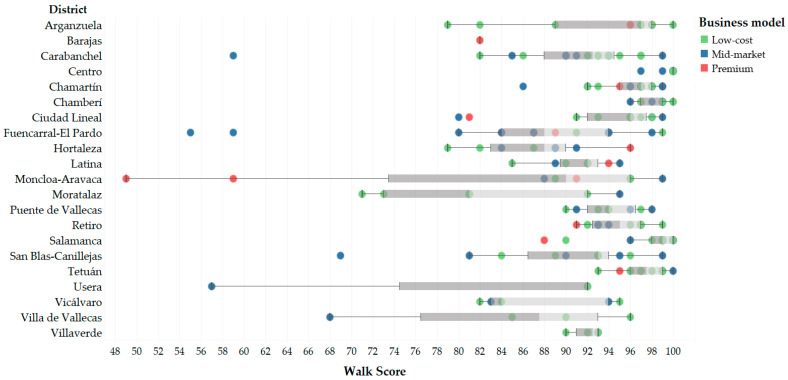
Fitness centers’ distribution by district.

Data from 193 fitness centers were retrieved (Figure 2). A representative distribution in all the areas of the municipality was ensured, including centers in the 21 districts of the city of Madrid, Spain (Figure 3).

Table 1 shows the means of Walk Score^®^, monthly fee, and opening hours. These results are presented according to the business model (low-cost, mid-market, and premium). An average Walk Score^®^ of 92.02 (SD = 9.02) out of 100 was determined. The average fee per month was 41.46 (SD = 28.70), and the average daily opening hours were 15.36 (SD = 3.16). Additionally, 20.20% of the centers have a swimming pool.

### 3.2. Association Between Walk Score^®^ and the Monthly Fee

**Figure 4:**
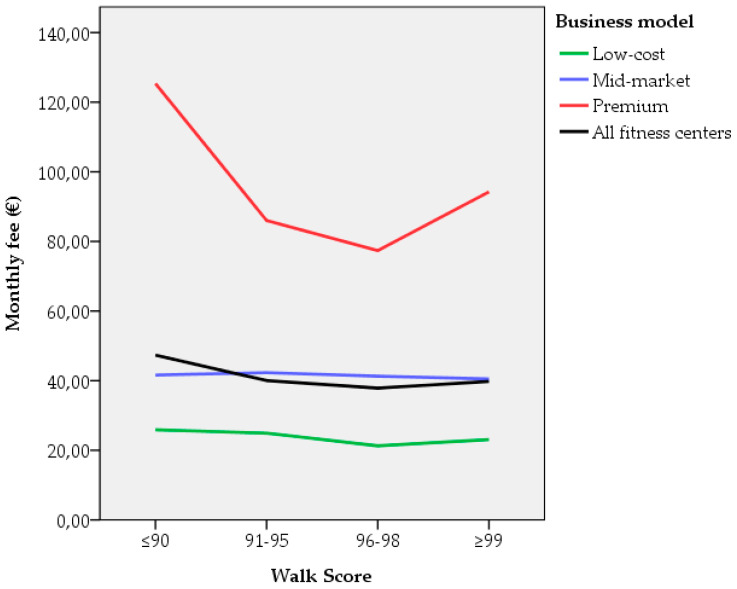
Monthly fee according to Walk Score^®^.

A nonlinear relationship between Walk Score^®^ and the monthly fee was obtained. According to the business model, no correlation was observed in either the low-cost or mid-market centers. Nevertheless, after measuring a normal distribution of data, the *t*-test only established statistically significant differences in premium centers, with lower fees in the range of 96–98 in contrast to a ≤90 Walk Score^®^ (Table 2). These data are presented graphically in Figure 4. No statistically significant differences were observed between other Walk Score^®^ ranges.

A regression analysis of Walk Score^®^ was conducted, following a curve estimation procedure. Different curves were estimated, namely, linear, quadratic, cubic, growth, and exponential. The quadratic model received the highest R-square (Table 3). Nevertheless, the analysis reveals a weak relationship between Walk Score^®^ and the monthly fee (*R*^2^ = 0.05, *F* (2, 190) = 5.27, *p* = 0.006). Therefore, medium levels of Walk Score^®^ (96–98) weakly contribute to lower fees.

### 3.3. Association Between Walk Score^®^ and Opening Hours

The Mann–Whitney U test was performed to compare the average opening hours between the four Walk Score^®^ groups (Table 4). The results show statistically significant differences between the ranges of ≤90 and 96–98, as well as between the groups of 91–95 and 96–98. Therefore, medium levels of Walk Score^®^, specifically between 96 and 98, are associated with wider opening hours.

### 3.4. Association Between Walk Score^®^ and the Existence of a Swimming Pool

The Mann–Whitney U test was also conducted to compare the average Walk Score^®^ between fitness centers with a swimming pool and centers lacking a swimming pool. The results show statistically significant differences when comparing both clusters. Therefore, a lower Walk Score^®^ is associated with swimming pools in the fitness centers (Table 5).

## 4. Discussion

Walkability is an important concept in urban planning, with great implications for the population, since walkable places are usually related to economic performance, including real estate development and values as a result of their attractiveness to permanent and temporary populations [7,36]. This paper deals with an innovative approach for measuring the relationship between walkability and specific variables of fitness centers (price, opening hours, and aquatic services) as fundamental services for physical activity promotion, which help to improve citizens’ health.

Firstly, regarding the association between Walk Score^®^ and the monthly fee of fitness centers, a weak nonlinear relationship was observed, meaning that there is not a strong association between the two variables. These results show coherence with previous work on tourist attractions and walkability [14]. However, our results contrast with research on tourist accommodations. Although hotels and AirBnB locations also showed a nonlinear relationship, the range of 93–96 Walk Score^®^ displays higher prices [23]. This weak link between walkability and price has implications for sports centers and other leisure-oriented businesses.

For the aforementioned reasons, there is no direct correlation between fitness centers with a higher Walk Score^®^ and higher fees. Only in premium centers, a certain degree of association is proven. When analyzing premium centers, a quadratic equation curve fits in the model, as was presented in previous research [14]. Thus, fitness centers with higher fees are often located in less walkable locations. This circumstance may be related to the specific features of the fitness industry and the diverse business models, together with the preferences and needs of the users of sports centers, since as was observed in previous studies, price and location are among the criteria of greatest weight for users in Spain [37], the United States, and Canada [38]. Especially for fitness centers with a low-cost model, service convenience (where walkability would be integrated) is tightly related to user perceived quality, user satisfaction, and client loyalty [39]. In fact, regarding fitness centers and their locations, previous studies have shown that clients are willing to commit to even a 30 min commute time to the facility if the perceived quality is better [39], while a 15 min commute time positively influences the client’s adherence to the center, leading to the client’s involvement in a longer membership and longer member continuity [37,40,41]. This is especially important for increasing levels of physical activity and, subsequently, impacts citizens’ health.

A good explanation for why users are willing to walk longer or to travel longer distances for premium services could be related to perceived service quality and added value. Previous studies have proven that the enjoyment of positive customer experience is associated with higher engagement levels, recommendation rates, and membership renewal intentions, with positive effects on all these variables [42,43,44,45]. For that reason, customers of premium fitness centers could be willing to walk longer distances or travel to less walk-friendly areas. This phenomenon has also been detected in the tourism sector [2,46] since tourists are willing to walk longer distances to locations with a lower Walk Score^®^ in order to enjoy the most popular tourist attractions.

Secondly, regarding the relationship between opening hours and Walk Score^®^, our findings show that a moderate to high Walk Score^®^ (96–98) correlates with longer opening hours. There is no direct correlation between fitness centers with a higher Walk Score^®^ and wider opening hours. Good management practices encourage sport facilities managers to reinforce their company strengths [32]. Therefore, for fitness centers whose location is considered a strength, maximizing and enlarging opening hours contributes to maximizing this strength. Identical to the comparison between Walk Score^®^ and price, low-cost centers need to pursue high service convenience, where opening hours may be influential, achieving ultimately a good perceived quality, user satisfaction, and client loyalty [38].

Lastly, regarding the relationship between the existence of a swimming pool at the gyms and Walk Score^®^, our findings show that a lower Walk Score^®^ correlates with the existence of aquatic services. In this regard, the ability to enjoy guided and free aquatic physical activities is an attractive aspect for gym users, especially affecting client satisfaction in the case of group class swimmers and future intentions in the case of free swimming users [47]. It has been shown that a swimming pool is not considered essential, but influential and attractive for customers at the moment of gym enrolment, even if swimming pool use rates are low [37]. However, the swimming pool is not always a profitable space, especially for fitness centers whose main focus is not related to aquatic activities [40]; therefore, gyms located in better locations (i.e., with a more expensive rental or surface fee) prefer not to dedicate a big surface area to these aquatic services.

## 5. Conclusions

This paper contributes to understanding the association between the geographical distribution of fitness centers and the variables of price, opening hours, and aquatic services. Firstly, no direct correlation is shown between Walk Score^®^ and monthly fee. A weak quadratic model is followed only by premium centers, with higher prices in less walkable areas, meaning that members of these centers are willing to travel to less walk-friendly areas. Secondly, the association between Walk Score^®^ and opening hours is not totally confirmed. Fitness centers in moderately to highly walkable locations tend to widen their average hours of operation, but there is not a direct correlation. Thirdly, the existence of a swimming pool is associated with a lower Walk Score^®^. In this regard, fitness centers in less walkable areas try to offer additional services such as aquatic services to differentiate themselves from better located competitors. However, centers in walkable locations do not need to invest in swimming pools and prefer to use this area for other purposes. All these data are relevant for increasing the adherence to fitness services. A better tailored experience would help to promote physical activity services participation and engagement.

This research has clear practical implications, mainly for managers of sports services. Business location is particularly important for increasing levels of adherence to fitness centers, helping to increase levels of physical activity. Understanding the geographical distribution of fitness centers would help them to tailor the offer to potential customers. Walk Score^®^ can be a useful open resource for managers of sports services at the time of deciding the best location and fitness center characteristics.

However, this work deals with the limitation of the local features of the municipality of Madrid. The urban distribution of Madrid shares many characteristics with other European capitals. However, our conclusions could be limited by specific local conditions and particular issues of Madrid. Nevertheless, there is no evidence in recent previous literature [19,20,21] to believe that Walk Score^®^ substantially differs outside the United States, mainly in high population density areas. Despite the applied approach, we recognize that there are other approaches, especially regarding spacial autocorrelation. Future studies should address different cities for further examination of fitness center parameters’ geographical distribution. Additionally, more extensive areas could be assessed, including both urban and rural spaces.

## Figures and Tables

**Table 1:** Descriptive statistics.

Variable	All Cases (SD)	Low-Cost (*n* = 84)	Mid-Market (*n* = 82)	Premium (*n* = 27)
Walk Score^®^	92.02 (9.02)	92.71 (6.35)	91.82 (10.26)	90.44 (11.83)
Monthly fee (€)	41.46 (28.70)	23.88 (4.39)	41.40 (7.47)	96.33 (41.48)
Daily opening hours	15.36 (3.16)	15.20 (1.74)	15.32 (3.88)	15.97 (4.05)

**Table 2:** Monthly fee *t*-test in premium centers according to Walk Score^®^.

Walk Score^®^	Average Monthly Fee (SD)	Difference	*p*-Value
≤90	125.37 (48.74)	47.99	0.032
96–98	77.38 (25.19)		

**Table 3:** Regression curve estimation.

Equation	Model Summary					Parameter Estimates		
	R-Square	F	df1	df2	Sig.	Constant	b1	b2
Quadratic	0.053	5.271	2	190	0.006	276.248	−5.142	0.028

**Table 4:** Opening hours comparison according to Walk Score^®^ groups.

Walk Score^®^	Average Opening Hours (SD)	Difference	*p*-Value
≤90	14.88 (3.14)	0.83	0.036
96–98	16.25 (3.44)		
91–95	14.74 (2.50)	0.97	0.040
96–98	16.25 (3.44)		

**Table 5:** Walk Score^®^ comparison in fitness centers with and without a swimming pool.

Swimming Pool	Average Walk Score^®^ (SD)	Difference	*p*-Value
No	93.32 (7.75)	6.47	0.000
Yes	86.85 (11.61)		
